# Research on Drugs and Vaccines for COVID-19 Should Be Conducted and Published With Caution

**DOI:** 10.2188/jea.JE20200408

**Published:** 2020-12-05

**Authors:** Fei Chen, Shuai Gong, Run-Qian Li

**Affiliations:** 1 Department of Physiology, Jining Medical University, Jining, China; 2 Department of Animal Genetics Breeding and Reproduction, Shandong Agricultural University, Tai-an, China

Developing effective drugs and vaccines is urgent for ending the coronavirus disease 2019 (COVID-19) pandemic. However, controversies emerged with the feverish search for them, and a few papers have been withdrawn or retracted, such as research on chloroquine/hydroxychloroquine,^1^^–^^3^ vitamin D,^4^ lopinavir/ritonavir,^5^ and favipiravir.^6^ In several vaccine clinical trials, solicited adverse events that occurred in some volunteers included fatigue, headache, chills, and muscle ache. Hence, several vaccine trials have now been halted.^7^^–^^9^ The conflicting results and the crisis of confidence resulted from article retractions and side effects after vaccination may interfere with the doctors’ medical decision-making, intensify the anxiety over the disease among the general public, and even lead to arbitrary drug use. In this case, the effects of drugs and vaccines need to be adequately clarified with more responsible attitudes. Dinis-Oliveira (2020) has pointed out that all players involved must attend specific educational programs and that a “National Agency for Scientific and Academic Integrity” should be created during the COVID-19 pandemic.^10^ In the meantime, we would like to propose several feasible suggestions for researchers and journals.

Although the rapid spread of COVID-19 has altered the traditional publication procedure of papers, researchers must abide by the principles of scientific research unswervingly. First of all, they need to avoid “political interference”. The conclusion of their research should be based on the real, objective, and verified data rather than the specific point of view or hypothesis. Meanwhile, exaggerating the study results for the sole purpose of publication or sensation should never be advocated. Second, scientists should conduct randomized double-blind controlled studies with large sample sizes, as before. Moreover, the potential damage to other organs or whether there will be any sequelae after the treatment needs to be considered when evaluating the efficacy of the drug or vaccine. Third, unprecedented attention should be paid to the communication and cooperation among those who are studying the same drug regimen. In addition, it is not appropriate to announce the results to the news media before the paper is finally accepted.

As for the journals, considering the difficulty in organizing massive duplicate tests or recruiting editors who are adept at analyzing data, we would like to propose the following suggestions. (1) The manuscripts on COVID-19 should be reviewed more strictly. Authors need to provide more detailed information such as data source and collection process. (2) In order to improve the detection efficiency and accuracy of the manuscripts, journals could consider sending them to a company using artificial intelligence for testing. (3) When reviewing COVID-19 papers, the number of peer reviewers could be appropriately increased, and at least one data analyst should be invited to examine the validity of the data in experiments. In the meanwhile, editors should avoid choosing reviewers suggested by authors to make peer review more objective and rigorous. (4) Replication studies and null results of drugs or vaccines on COVID-19 are also meaningful. Therefore, journals should keep a neutral attitude towards them for publication. (5) If the subject of the article is a drug or vaccine that has been seriously controversial, the journal could ask for the authors’ permission and then release it in advance, accept the review by scientists worldwide, and note that “the article has not been accepted and the conclusion is yet to be reviewed.”
